# A modified and automated version of the 'Fluorimetric Detection of Alkaline DNA Unwinding' method to quantify formation and repair of DNA strand breaks

**DOI:** 10.1186/1472-6750-9-39

**Published:** 2009-04-23

**Authors:** María Moreno-Villanueva, Ragen Pfeiffer, Thilo Sindlinger, Alan Leake, Marcus Müller, Thomas BL Kirkwood, Alexander Bürkle

**Affiliations:** 1 Lehrstuhl Molekulare Toxikologie, Fachbereich Biologie, Universität Konstanz, D-78457 Konstanz, Germany; 2 Institute for Ageing and Health, Henry Wellcome Laboratory for Biogerontology Research, Newcastle University, Campus for Ageing and Vitality, Newcastle upon Tyne NE4 5PL, UK; 3 Abteilung Tumorvirologie, Deutsches Krebsforschungszentrum, D-69120 Heidelberg, Germany; 4 TherapySelect GmbH & Co. KG, Im Neuenheimer Feld 584, D-69120 Heidelberg, Germany

## Abstract

**Background:**

Formation and repair of DNA single-strand breaks are important parameters in the assessment of DNA damage and repair occurring in live cells. The 'Fluorimetric Detection of Alkaline DNA Unwinding (FADU)' method [Birnboim HC, Jevcak JJ. Cancer Res (1981) 41:1889–1892] is a sensitive procedure to quantify DNA strand breaks, yet it is very tedious to perform.

**Results:**

In order (i) to render the FADU assay more convenient and robust, (ii) to increase throughput, and (iii) to reduce the number of cells needed, we have established a modified assay version that is largely automated and is based on the use of a liquid handling device. The assay is operated in a 96-well format, thus greatly increasing throughput. The number of cells required has been reduced to less than 10,000 per data point. The threshold for detection of X-ray-induced DNA strand breaks is 0.13 Gy. The total assay time required for a typical experiment to assess DNA strand break repair is 4–5 hours.

**Conclusion:**

We have established a robust and convenient method measuring of formation and repair of DNA single-strand breaks in live cells. While the sensitivity of our method is comparable to current assays, throughput is massively increased while operator time is decreased.

## Background

Formation and repair of DNA single-strand breaks in live cells is an important functional parameter in the assessment of genotoxicity, and therefore the reliable and convenient assessment of DNA strand breakage is of crucial importance for a wide range of basic and translational biological research fields including genetic or environmental toxicology (mechanistic research; screening for genotoxins); DNA repair research (basic biochemical studies; molecular epidemiology); cancer research; pharmacology and drug development; ageing research and many more. The measurement of DNA strand breaks by FADU as described by Birnboim & Jevcak [1] is based on the partial denaturation ("unwinding") of double-stranded DNA under controlled alkaline conditions. Briefly, after infliction of DNA damage, cell lysis was performed. DNA strand breaks are sites where controlled unwinding of DNA can start under controlled conditions of pH and temperature. To terminate unwinding, a neutralising solution was added. To quantify the amount of DNA remaining double-stranded after the alkali incubation, ethidium bromide was added as a fluorescent probe. Low fluorescence intensities indicated a large number of DNA strand breaks present at the time of lysis. In practical terms, the following samples are processed in parallel: *T samples *are representative of the total amount of DNA present and are obtained by adding neutralisation solution prior to the alkaline unwinding solution. As a consequence, the critical alkaline pH needed for DNA denaturation will never be reached and no unwinding will occur. On the other hand, *P*_0 _*samples *do undergo alkaline unwinding at the ends of the chromosomes, at endogenous DNA strand breaks and at replication forks, thus reflecting physiological conditions. By contrast, in *B samples *DNA will denature completely due to the massive induction of DNA breaks, *e.g. *by sonication or shearing, and therefore only background fluorescence is observed. *P*_*x *_*samples *(*P*_1_, *P*_2_, *P*_3 _*etc*) are samples in which to measure DNA damage-induced DNA strand breaks, and *R*_*x *_*samples *(*R*_1_, *R*_2_, *R*_3 _*etc*) are samples where cells are post-incubated to allow repair.

Since the original publication by Birnboim & Jevcak [1], a number of assay modifications have been described that led to reduction of the numbers of cells needed and faster completion of the assay [2-7]. The modified and automated version of the FADU assay described in the present paper enables measurement of DNA strand breaks and DNA repair in a very reliable and convenient manner by exploiting the power of a liquid handling device (LHD) in terms of its extremely high level of control of various parameters and the reproducibility of dispensing small volumes. In particular the transfer at sub-millimetre precision within the three dimensions of the LHD's workspace as well as precise control of timing and rate of liquid delivery are extremely useful features, as the creation of a separate layer of alkaline solution on top of the lysate without any mixing is critical [*cf*. [1]]. Protecting the samples from light by encasing the LHD also greatly adds to assay sensitivity, as the genomic DNA liberated from histones by the urea present in the lysis buffer is prone to DNA breaks induced artificially by visible light.

## Methods

### Plasmid

For a set of pilot experiments we used the plasmid pEGFP-N1 (size: 4.7 kb; BD Biosciences, Heidelberg, Germany). The plasmid was restricted or not with *Eco*RI (NEB, Frankfurt am Main, Germany). To obtain linear DNA, 8 μg of plasmid DNA was digested in 60 μl distilled H_2_O, 8 μl 10× buffer and 4 μl *Eco*RI at 37°C for 1 h. To stop digestion the plasmid solution was incubated at 65°C for 20 min. Completeness of the digestion was verified by agarose gel electrophoresis.

### Primary cells

Ethical clearance for obtaining peripheral venous blood from healthy adult volunteers was obtained from the University of Konstanz Ethics Committee. Peripheral blood mononuclear cells (PBMC) were purified from freshly drawn blood. All donors were healthy adult volunteers. For the separation of PBMC we used Percoll (Pharmacia Biotec AB, Uppsala, Sweden) gradient centrifugation. Briefly, 7 ml of whole blood was drawn with heparin as anti-coagulant. The blood was diluted 1:2 with PBS, layered on the top of 15 ml Percoll solution and centrifuged at 800 *g *for 10 min at room temperature in a swing-out rotor without break. Mononuclear cells harvested from the gradient were washed by resuspension in PBS and centrifugation at 200 *g *for 10 min at 0°C.

### Cell lines

Jurkat T cells were cultured in suspension in tissue culture flasks (Corning, Schiphol-Rijk, The Netherlands) in RPMI medium (Sigma-Aldrich, Deisenhofen, Germany) supplemented with 10% FCS and antibiotics (100 U/ml penicillin, 100 μg/ml streptomycin).

HeLa cells were cultured as monolayer in non-pyrogenic tissue culture flasks (Corning, Schiphol-Rijk, The Netherlands) in DMEM medium (Sigma-Aldrich, Deisenhofen, Germany) supplemented with 10% FCS and antibiotics. Prior to any analyses by FADU assay, monolayers were trypsinized, and detached cells were resuspended in complete medium (DMEM plus FCS plus antibiotics), followed by centrifugation at 200 *g *for 10 min. Pellets were resuspended in DMEM without FCS (see below, *3 – DNA Repair*).

### 96-well plate

A 96-well plate master block (2 ml) with V-shaped bottom (Greiner-Bio One GmbH, Frickenhausen, Germany) was used, which was cut horizontally to hold 1 ml in order to enable measurement of the fluorescence intensity in a commercial fluorimeter for standard plates.

After use, the 96-well plates were washed with 1 M NaOH for 1 h, rinsed with water, incubated in DMSO (Merck, Darmstadt, Germany) for 5 min, once again rinsed with water and finally washed in 70% ethanol for 5 min.

### Liquid handling device and accessories

For the automated steps of the new FADU protocol (see below) a TECAN Genesis RSP 100 liquid handling device (LHD) from TECAN AG (Hombrechtikon, Switzerland), controlled by GEMINI V4.0 software from TECAN software GmbH (Berlin, Germany) was used.

For the precise positioning of the cells in the X-irradiation device, a custom-made ice box, made of plastic and comprising an aluminium mould, was used. It can accommodate a 96-well-plate or Eppendorf tubes and maintains the samples at 0°C. This box can also be inserted at a defined position in the working area of the LHD to allow transfer of the samples to a custom-made cooling device mounted in the LHD's working space. The cooling device accommodates a 96-well plate and is connected to a cooling water bath for temperature control.

The eight nozzles of the LHD find their target with regard to X, Y, Z coordinates with a precision of 0.2 mm. Solutions to be transferred by the LHD are stored in trays, and those for ice-cold solutions are placed in a temperature-controlled holder. All buffers are accessible to the nozzles from the top. For protection against light the LHD is accommodated in a closed box with a roller shutter at its front.

### Steps of the modified and automated FADU protocol

**Figure 1:**
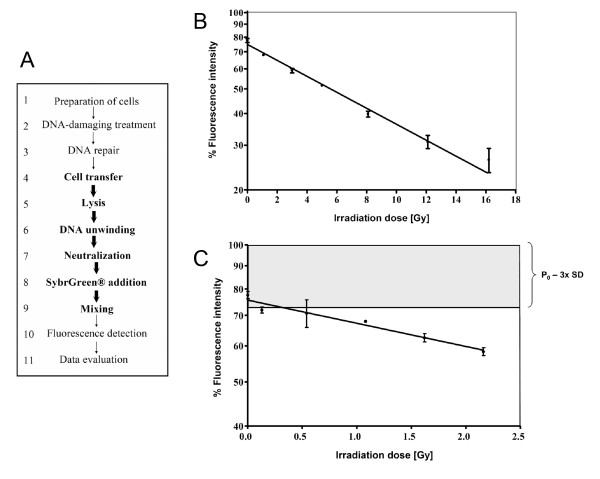
**(A) Overview of the essential steps of the modified and automated FADU assay**. After preparation of the cells, DNA damage is inflicted, followed by further incubation to allow DNA repair. The automated steps are highlighted in bold. **(B)**, **(C) **Dose-dependent induction of DNA strand breaks by X-rays in human PBMC (**B**, high dose range; **C**, low dose range). PBMC were exposed to various doses between 0 and 16.2 Gy. Note that the percentage of unwound DNA increased with increasing doses. Data are presented as means ± SDs from 3 independent experiments performed on PBMC from different donors. The detection threshold was set as mean minus 3 SD of the unirradiated controls (P_0_).

**Figure 2:**
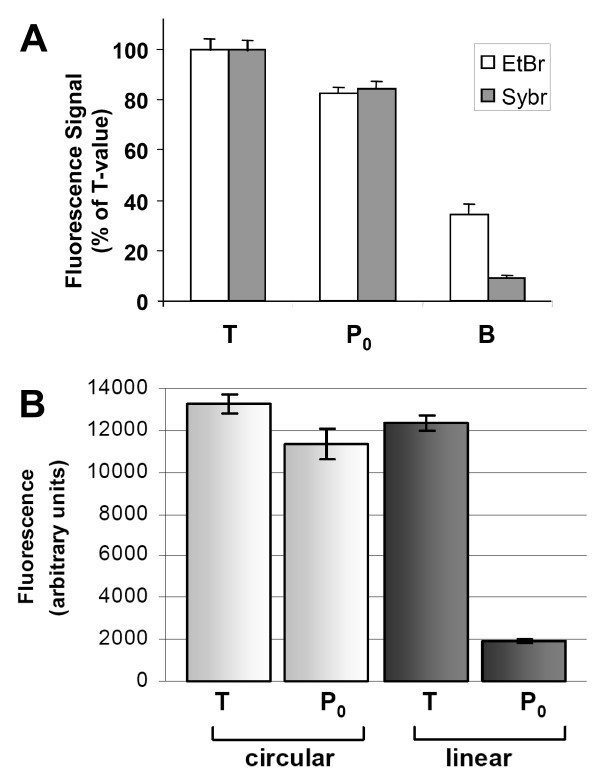
**(A) Comparison of SybrGreen (Sybr) with ethidium bromide (EtBr) as a fluorescent probe for the FADU assay**. For each experimental point in this experiment, 80,000 human PBMC were suspended in 70 μl suspension buffer at 0°C. For B samples, 70 μl of lysis buffer was added and the mixture was sheared by 20 passages through a 0.5-mm cannula, followed by transfer to a 96-well plate [140 μl per well]. For P and T samples 70 μl of cell suspension was transferred per well and 70 μl lysis solution was added at 0°C. This and all subsequent pipetting steps were performed by the LHD. After 12 min of lysis, 70 μl of unwinding solution was added on the top of the cell lysate followed by incubation at 15°C for 90 min. To stop DNA unwinding, neutralisation solution was added. Then 150 μl of the mixture was combined in plastic cuvettes with 500 μl of either Sybr or EtBr solution. Fluorescence detection was done at excitation 480 nm and emission 520 nm. **(B) **Alkaline unwinding of plasmid DNA as a model substrate. The circular form of the plasmid (white) could not be unwound and retained nearly 100% of the total fluorescence (T circular). The P_0 _values of the linear form (grey) represent unwound DNA and display less than 20% double/stranded DNA compared to non-denaturing control conditions (T linear). To obtain the T values, neutralisation solution was added before the alkaline solution. Error bars represent SDs from 8 replicates.

This new version of the FADU assay comprises a total of 11 steps described below. It should be noted that steps 2 and 3 are alternatives, depending on whether or not DNA repair is to be analysed. Steps 1, 2, 3, 10 and 11 were performed manually whereas the critical steps 4–9 have been automated (Fig. 1A). It should be noted that in some experiments performed for protocol optimisation (Fig. 2A), different conditions were used, as indicated.

*T samples *represent the total amount of DNA present.*B samples *represent the background fluorescence observed when DNA is denatured completely. *P*_0 _*samples *reflect DNA strand interruptions present under physiological conditions. *P*_*x *_*samples (P*_1_, *P*_2_, *P*_3 _*etc*) are from cells to which DNA damage had been inflicted in order to induce DNA strand breaks, and *R*_*x *_*samples *(*R*_1_, *R*_2_, *R*_3 _*etc*) are from cells allowed to repair DNA damage.

#### Steps 1 and 2 – Preparation of Cells and DNA Damage by Irradiation

For the analysis of DNA strand break formation only (without repair) cells were resuspended in suspension buffer (0.25 M *meso*-inositol; 10 mM sodium phosphate, pH 7.4; 1 mM magnesium chloride) or in medium without FCS. For the irradiation we used an X-ray generator from CHF Müller (Hamburg, Germany). The dose to be delivered was applied by variation of irradiation time at a fixed dose rate. The irradiation parameters were the following: 70 keV energy, 1.25-mm aluminium filter, and 9.4 mA current. The dose was measured using a PTW Universal Dosimeter UNIDOS E [D545.151.00/02] from PTW-Freiburg (Freiburg, Germany). Seventy μl of cell suspension (at 100 cells/μl) in a 96-well plate was irradiated on ice in a custom-made ice box described above.

#### Steps 3 and 4 – DNA Repair and Cell Transfer

For the assessment of DNA repair, cells were resuspended in DMEM without FCS or other supplements at a concentration of 6 × 10^5 ^cells per ml. Aliquots of 100 μl were cooled down in Eppendorf tubes and irradiated on ice. To allow DNA strand break repair, the damaged cells were incubated at 37°C for various time periods in an incubator. To stop repair 500 μl ice-cold suspension buffer was added to 100 μl cell suspension, respectively. Seventy μl/well of this cell suspension (*i.e. *7,000 cells) were transferred to the 96-well plate, which had been placed in the cooling device described above.

#### Step 5 – Lysis

The 96-well plate holding the 70 μl cell samples was positioned in the working space of the robot and kept at 0°C in the dark. Then automated dispensing of 70 μl of lysis solution (9 M urea; 10 mM NaOH; 2.5 mM cyclohexyl-diamine-tetraacetate; 0.1% sodium dodecyl sulphate; kept at RT) to each sample at a rate of 150 μl/s was initiated.

#### Step 6 – Alkaline solution

After 12 min of lysis (temperature of cooling device set to 0°C), 70 μl of alkaline solution (0.425 parts lysis solution in 0.2 M NaOH, pre-cooled to 0°C) was added on the top of the cell lysate in such a way as to form a second layer, thus avoiding any mixing with the lysate. To do this the nozzles of the LHD were positioned precisely 1.5 mm above the level of the lysate and the alkaline solution was dispensed at the very low rate of 10 μl/s. Then diffusion of alkaline solution into the lysate was allowed to occur for 15 min. Temperature readings showed that the content of the microwells displayed approximately 8°C during this time period. A pH value of 12.5 was reached under these conditions. Thereafter the temperature setting of the cooling device was shifted to 30°C and kept constant for 60 min in order to allow alkaline unwinding of the DNA.

#### Step 7 – Neutralization solution

Prior to the addition of 140 μl of neutralization solution (14 mM β-mercaptoethanol; 1 M glucose) at a rate of 200 μl/s the temperature was shifted to 22°C. For T-samples (see below), an internal standard representing cells with 100% double-stranded DNA, 140 μl of neutralization solution was added prior to the alkaline solution.

#### Steps 8 and 9 – SybrGreen^® ^addition and mixing

To determine the amount of double-stranded DNA, SybrGreen (10,000×) from MoBiTec (Göttingen, Germany) was used. After dispensing 156 μl diluted SybrGreen (1:8,333 in H_2_O), respectively, the samples were mixed by pipetting a volume of 400 μl up and down once at a rate of 100 μl/s.

#### Steps 10 and 11 – Read-out and interpretation of data

Samples were analysed in a 96-well-plate fluorescence reader at 492 nm excitation/520 nm emission immediately after SybrGreen addition. Statistical significance was evaluated by using Student's *t*-test. In some experiments B-samples were included as an internal standard representing lysates with no double-stranded DNA left after alkali exposure as a result of extensive DNA breakage induced by passing the lysate 20 times through a 0.5 mm cannula. In course of the work it was noted that, using SybrGreen as a fluorescent probe, there was a stable ratio between T and B values, and since the B values were quite low, B samples were omitted in the final FADU protocol.

## Results

### Optimisation of the FADU protocol

In the original FADU protocol, Birnboim & Jevcak had used ethidium bromide as a fluorescent probe to quantify the amount of DNA that remained double-stranded under the experimental conditions chosen [1]. When establishing our modified protocol, we first compared SybrGreen, a more recently developed fluorescent compound with high specificity for double-stranded DNA, with ethidium bromide. Our data indeed showed that SybrGreen produced much less background fluorescence (Fig. 2A) and thus increases assay sensitivity. Therefore for all subsequent analyses SybrGreen was used.

To characterise further the alkaline unwinding process we used purified DNA of plasmid pEGFP-N1, which was linearised or not with *Eco*RI, as a model substrate. While covalently closed circular plasmid DNA did not undergo unwinding under our assay conditions as revealed by the high P_0 _value, the linear form of the plasmid could unwind starting from its ends. Our data showed that only about 20% of DNA remained double-stranded (Fig. 2B). Agarose gels revealed that undigested plasmid preparations typically comprised a small fraction of nicked circular DNA (data not shown). This form of DNA can unwind under the conditions chosen, and this explains the slightly lower fluorescence signal of the circular DNA P_0 _samples compared to T values.

**Figure 3:**
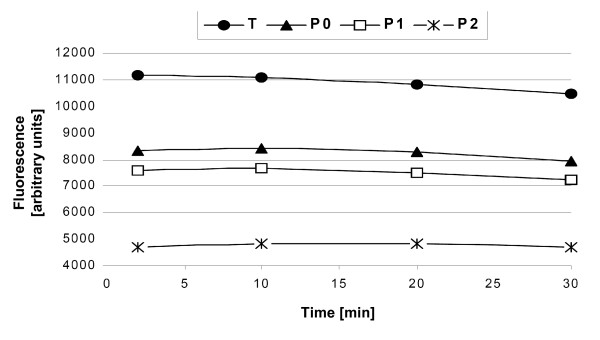
**Measurement of fluorescence signals at various time points after SybrGreen addition**. PBMC were exposed to an X-ray irradiation dose of 0.47 Gy (P_1_) or 2.3 Gy (P_2_). Cells in T and P_0 _samples were not irradiated. Note that the fluorescence signal decreased with increasing irradiation dose, as expected. Fluorescence intensity during the first 20 min upon SybrGreen addition remained constant but decreased thereafter. The ratios P_0_/T, P_1_/P_0 _and P_2_/P_1 _remained constant over the whole observation period.

To assess whether the time interval between SybrGreen addition and fluorescence reading is critical, we systematically measured fluorescence as a function of time after SybrGreen addition. All the samples showed saturation immediately after addition of SybrGreen. The fluorescence signal remained constant during 20 min. We conclude that the fluorescence determination yields stable results if readings are performed within 20 min after SybrGreen addition (Fig. 3).

In order to determine the minimal number of cells required, we irradiated cells at three different cell titres. The ratio of fluorescence signals of P_0_/T and P_1_/P_0 _using 297, 132 and 90 cells/μl varied less than 2.4% and of P_2_/P_1 _less than 7%, indicating that the automated FADU assay yields robust results over a range of cell titres. Interestingly, standard deviations tended to be smaller with lower cell titres (Table 1).

**Table 1:** Influence of cell titre on fluorescence signal intensity.

**Cells/μl**	**Ratios of fluorescence signal intensities**			**Standard deviation [%]**			
	**P_0_/T**	**P_1_/P_0_**	**P_2_/P_1_**	**T**	**P_0_**	**P_1_**	**P_2_**
297	0.82	0.94	0.77	5.5	10.6	12	12.7
132	0.84	0.96	0.68	3.3	4.5	4.9	5.4
90	0.86	0.95	0.71	2.9	4.6	4.2	3.2

P_1 _and P_2 _samples were irradiated with 0.47 or 2.3 Gy, respectively. Standard deviation was evaluated for 8 replicates, respectively, and expressed as % of mean values.

### Definitive assay protocol

The essential steps of the definitive assay protocol for the modified automated FADU assay are summarised in Fig. 1A and the detailed protocol is described in Materials and Methods.

To assess assay reproducibility we measured the dose-dependent unwinding induced by X-irradiation of cells in several independent experiments. DNA unwinding increased with increasing radiation dose in all experiments, and the results were highly reproducible, both in a high (Fig. 1B) and in a low irradiation-dose range (Fig. 1C). The lowest dose detected with statistical significance was 0.13 Gy (Fig. 1C).

**Figure 4:**
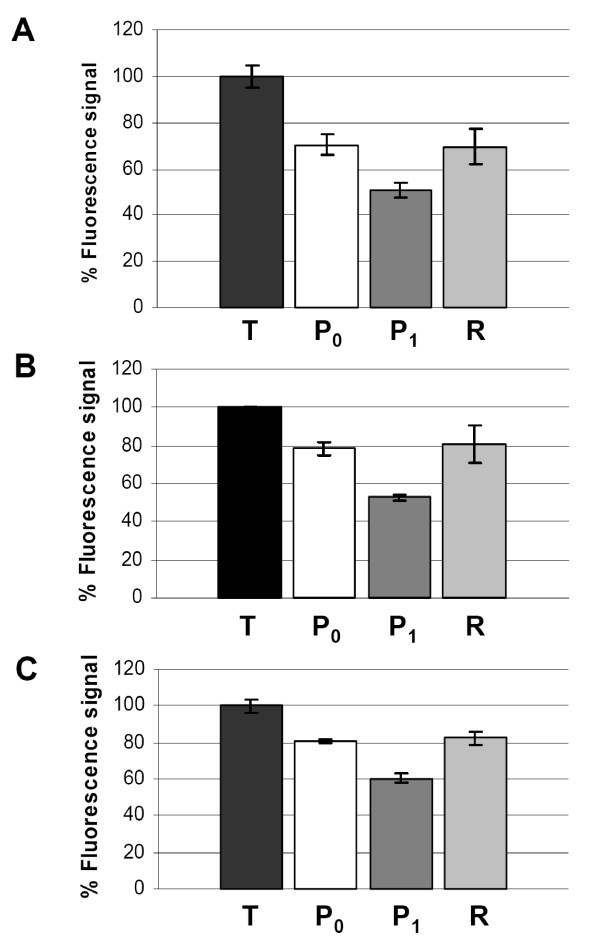
**DNA damage and repair in several different cell systems**. HeLa S3 cells (**A**), Jurkat cells (**B**) and human PBMC (**C**) were damaged with 2.3 Gy of X-irradiation (P_1_). To allow repair, cells were incubated at 37°C for 40 min (R). T and P_0 _samples were controls. In all cases the fluorescence signal of the P_1 _samples was lower that the control value P_0_, and those of the R values higher than the damaged samples P_1_. Error bars represent SDs from 8 replicates.

**Figure 5:**
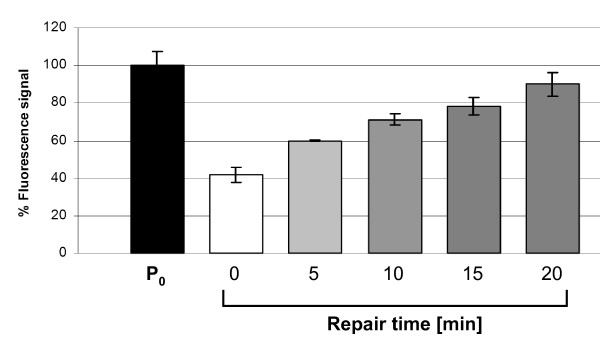
**Time course of DNA strand break repair of Jurkat cells in intervals of 5 min**. Cells were X-irradiated on ice with 6.8 Gy (basal line). To measure DNA strand break repair, the damaged cells were incubated at 37°C for different time periods as indicated (grey columns). The black column represents level of SybrGreen fluorescence obtained in undamaged cells (P_0 _values). Error bars represent SDs from 3 replicates.

We also tested the applicability of the automated FADU assay in several different cell systems. Induction of DNA strand breaks and repair were measured in PBMC and two human cell lines, *i.e. *the T-cell derived cell line Jurkat and the cervical carcinoma cell line HeLa. To measure DNA repair, the damaged cells were post-incubated at 37°C for 40 min. During this time period cells were able to repair the DNA breaks to the level of P_0 _samples, as is apparent from the recovery of SybrGreen fluorescence (Fig. 4). Analysis of the time course of repair in 5-min intervals revealed the steady progression of repair with time (Fig. 5).

## Discussion

A sensitive, robust and convenient assay for the detection and quantification of DNA damage and repair is useful not only for basic scientific research on DNA damage and DNA repair, but also for medical diagnostics (for example to assess individual genotoxic exposures and biological responses) and toxicological screening in the chemical and pharmaceutical industry.

The two current methods to measure DNA damage and repair with a high sensitivity are the "comet" assay and the conventional, manually performed FADU [1]. Detection limits of 0.03 Gy for the comet assay and 0.1 Gy for the FADU assay have been reported [8]. The principles of these two kinds of assays differ. The comet assay is based on different migration velocities of DNA fragments of different sizes in agarose gels. In an electrical field, fragmented DNA migrates out of the nucleus into the agarose gel and can be visualised by staining with a fluorescent dye. Viewed microscopically, the combination of the DNA that has stayed in the nucleus and the "tail" of DNA that has migrated makes the cells look like a "comet". The length and intensity of the comets in relation to the signal of the non-migrating nuclear DNA compared to the controls can be quantified with the help of software. Being based on a microscopic readout, the comet assay can also detect heterogeneity within a cell population, which is not possible with any lysate-based assay including FADU. The comet assay is, however, very tedious and requires a large number of steps to be performed by highly trained personnel. Likewise, the FADU assay in its original, manually operated version [1] is technically very demanding, labour-intensive and requires large numbers of cells.

The automated version of the FADU assay described here relies on a properly configured LHD equipped with some custom-made accessories. It is operated in a 96-well format and is able to measure DNA strand breaks and repair with a high reproducibility. We have been able to reduce the number of cells necessary for the assay dramatically. While Birnboim & Jevcak used 1–2 × 10^6 ^cells per data point [1], we were able to reduce the number of cells required by more than 100-fold to 4,900–8,400 cells. Temperature control is also part of the automated operation. Furthermore samples are completely protected from light throughout the duration of the automated steps. Using this system we have been able to measure DNA strand breaks and repair in a variety of cell systems. In our hands the lowest dose of X-ray-induced DNA strand breaks detected with statistical significance is 0.13 Gy. Collectively, the automated procedure including all the critical steps (Fig. 1A) takes 2 hours. Usually a complete FADU experiment for the assessment of DNA repair can be performed within 4–5 hours. While the FADU assay cannot detect cellular heterogeneity, determination of the average level of strand breaks will be sufficient for many purposes, and here the automated FADU is clearly superior due to its higher throughput and much lower labour utilisation.

Since the introduction of the comet assay, many modifications have been introduced to increase its performance [9] and enhance throughput [10-12]. A method using fluorescence analysis of DNA unwinding to measure DNA damage was also modified to decrease processing time [3,6]. However, the method described in the present paper is fully automated and has higher throughput than previous methods for measuring DNA damage and repair. It should be noted that we have typically used 8 replicates per T/P_0_/P_x_/R_x _value, respectively. Depending on the magnitude of the effect to be studied, however, statistically significant results can be obtained with only half the number of replicates (data not shown), thus further increasing throughput.

Another clear-cut advantage of the automated FADU assays compared with the comet assay is the better definition of time of lysis. While in the FADU assay lysis occurs instantaneously at the time of addition of lysis solution, this may not be the case in the comet assay as lysis solution has to diffuse through the soft agar, in which the live cells are embedded. This ill-defined situation is likely to decrease precision of the comet assay especially when assessing the rapid phase of DNA repair.

It is known that at pH values >13, alkali-labile sites are converted to DNA single-strand breaks and thus give rise to additional initiation sites for unwinding. Our pH measurements, however, showed that upon addition of the alkaline unwinding solution, the pH is 12.5, and therefore we have conditions that will not or only marginally generate strand breaks at alkali-labile sites [13].

## Conclusion

In conclusion, we have established a robust and convenient method for the quantitative assessment of formation and repair of DNA single-strand breaks in live cells. While the sensitivity of our method is comparable to the assays most often used currently, the throughput is massively increased while the operator time decreased.

## Competing interests

The authors declare that they have no competing interests.

## Authors' contributions

MMV and RP contributed to designing, performing, evaluating and interpreting the experiments shown in this paper. They also contributed to the set-up of the hardware and master protocol of the assay and to drafting the manuscript. TS, AL and MM performed in preliminary experiments and contributed to the set-up of the hardware and master protocol of the assay. TBLK contributed to directing the project and writing the manuscript. AB conceived and led the project. All authors read and approved the final manuscript.
